# Rotational and vibrational dynamics in the excited electronic state of deprotonated and protonated fluorescein studied by time-resolved photofragmentation in an ion trap

**DOI:** 10.1063/1.4953367

**Published:** 2016-06-08

**Authors:** Dimitri Imanbaew, Maxim F. Gelin, Christoph Riehn

**Affiliations:** 1 Fachbereich Chemie, Technische Universität Kaiserslautern, Erwin-Schrödinger-Str. 52-54, D-67663 Kaiserslautern, Germany; 2 Fakultät für Chemie, TU München, Lichtenbergstraße 4, D-85747 Garching, Germany; 3 Landesforschungszentrum OPTIMAS, Erwin-Schrödinger-Str. 46, D-67663 Kaiserslautern, Germany

## Abstract

Excited state dynamics of deprotonated and protonated fluorescein were investigated by polarization dependent femtosecond time-resolved pump-probe photofragmentation in a 3D ion trap. Transients of deprotonated fluorescein exhibit vibrational wavepacket dynamics with weak polarization dependence. Transients of protonated fluorescein show only effects of molecular alignment and rotational dephasing. The time resolved rotational anisotropy of protonated fluorescein is simulated by the calculated orientational correlation function. The observed differences between deprotonated and protonated fluorescein are ascribed to their different higher lying electronically excited states and corresponding structures. This is partially supported by time-dependent density functional theory calculations of the excited state structures.

## I. INTRODUCTION

Fluorescein (FL) and its derivatives represent one of the most popular categories of dyes in biochemistry. Due to their high molar absorptivity and fluorescence yield,^1^ fluorescein based markers are often used in fluorescence labeling^2,3^ or as probes for, e.g., redox cycles in living cells^4^ and sensing of various metals ions or other small molecules.^5^ In solution, FL appears pH-dependent in up to four prototopic forms (dianion [FL-2H]^2−^, monoanion [FL − H]^−^ as a phenolate or carboxylate, neutral [FL], and cation [FL + H]^+^) exhibiting specific absorption and emission characteristics.^1^ FL is easily chemically linked to biopolymers and therefore used in (steady-state) fluorescence anisotropy measurements,^6–8^ with the goal to improve microscopic fluorescence imaging^7–10^ and study the dynamics of protein-folding^11^ or conformational rearrangements by time-resolved fluorescence anisotropy (TR-FA).^12–14^ The importance of the latter applications, their perspective for Förster resonance energy transfer (FRET) studies,^15–17^ and furthermore, the search for insight into the influence of solvation on the different prototopic FL forms has sparked interest in gas phase studies coupled with mass spectrometric or fluorescence detection.^18–21^ In addition, recently, it was convincingly demonstrated that a gas-phase analogue to TR-FA is possible by implementation of a femtosecond time-resolved photodetachment anisotropy (TR-PA) scheme.^22^ In that report, rotational and vibrational wavepacket dynamics were observed for [FL-H]^−^ by velocity-map imaging detection of photoelectrons.^22^

**SCHEME 1.**
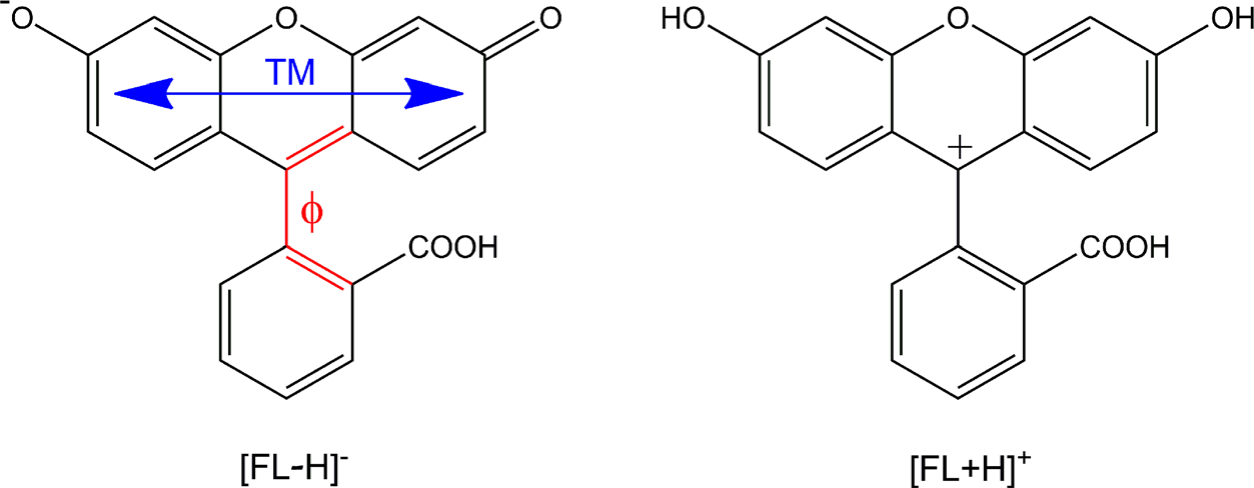
Schematic structure of phenolate [FL − H]^−^ (left) and [FL + H]^+^ (right). TM and φ denote the transition dipole moment for the S_1_ ← S_0_ transition of [FL − H]^−^ and the dihedral angle of the torsional coordinate of the benzoic acid ring vs. the xanthene unit, respectively.

In the present study, we extend these experiments by employing the complementary method of linearly polarized femtosecond transient photofragmentation (tPF) in an ion trap, which allows us to investigate the dynamics of the monoanionic phenolate [FL − H]^−^ and the cationic fluorescein species [FL + H]^+^ (Scheme 1). We present first results obtained by this technique and discuss differences with respect to the molecular structures of the involved electronically excited states of [FL − H]^−^ and [FL + H]^+^, as well as the utilized detection schemes (TR-PA vs. tPF) in studies of [FL − H]^−^.

## II. EXPERIMENTAL SETUP AND CALCULATIONS

The disodium salt of FL and methanol of LC-MS grade were purchased from Sigma-Aldrich and used without further purification. Time-resolved and polarization-dependent photofragmentation experiments were conducted using a modified Paul-type quadrupole ion trap mass spectrometer (amaZon speed, Bruker Daltonics) in combination with a Ti:sapphire oscillator and amplifier system (Wyvern 1000, KMLabs).^23^ Briefly, [FL−H] ^− ^and [FL + H]^+^ were generated by electrospray ionization of a methanolic solution of the FL salt (c = 5 × 10 ^− 6^ M) in negative and positive ion mode, respectively. For the study of [FL + H]^+^, a small amount of formic acid (1 vol. %) was added to the sample solution in order to increase the cation signal. The sample solution was continuously infused by a syringe pump at a flow rate of 120 *μ*l/h. Nitrogen as drying gas was set to a flow rate of 4 l/min at 180 °C. The nebulizer pressure was set to 5 psi (345 mbar).

The femtosecond laser pulses were generated in a cryogenic ultrafast regenerative laser amplifier system delivering 50 fs pulses at 1 kHz repetition rate (central wavelength of ca. 785 nm). The pulse train was split to pump two optical parametric oscillators (TOPAS-C, Light Conversion) for generating pump and probe pulses of tunable wavelength (240–2600 nm). Temporal delay between pump (520 nm, 0.3 *μ*J; 425 nm, 0.8 *μ*J) and probe pulses (1200 nm, 150 *μ*J) was controlled via an optical delay line. Pump and probe pulses were spatially overlapped quasi-collinearly by focusing into the center of the Paul ion trap with a lens (f = 50 cm). The beam diameter in the ion trap was estimated to be ∼1 mm using the knife-edge technique. The relative polarization of pump and probe pulses was controlled by a Berek compensator in the pump path. The initial 1 kHz repetition rate was reduced to ∼330 Hz by an optical chopper. Each isolated portion of ions was irradiated by 50 pump/probe pulse pairs.

The tPF signals were recorded as extracted ion chromatograms while continuously varying the delay between the pump and probe pulses. Evaluation of the transient signals was performed as F_i_/(F_i_ + P_j_), where F_i_ and P_j_ are the sums of the intensities of fragment and parent ion signals, respectively. To evaluate the temporal resolution at a given pump/probe wavelength combination, we recorded the multi-photon ionization signal of neutral furan in the ion trap as a function of time delay between the pump and probe pulses.^24^ The obtained signal represents an intensity cross correlation function (ccf) of the pump and probe laser pulses. The instrumental system response was then estimated from the FWHM of the resulting Gaussian-shaped photoionization signal. A typical value for the FWHM of the ccf at 520 nm (pump)/1200 nm (probe) [425 nm/1200 nm] is ∼110 fs [∼135 fs].

Time-resolved experiments with polarization-sensitive detection reveal reorientation of transition dipole moments (TMs) and relaxation of the optically induced anisotropy in real time, both in liquid phase^25^ and in the gas phase.^26^ The signals obtained from our polarization dependent tPF experiments arise from the same phenomena, as observed in TR-PA measurements. We therefore used the same method of analysis to disentangle rotational from population dynamics, as described and applied successfully in Ref. 22. Briefly, this analysis originates from TR-FA and is implemented by measuring transient signals at parallel and perpendicular relative linear laser polarization. This method allows us to calculate an isotropic signal *S*(*t*) unfettered by rotational dynamics, as well as an anisotropic function *r*(*t*), dealing with the directionality of the probe process with respect to pump photon absorption. *S*(*t*) and *r*(*t*) are defined as

$$S(t)=\frac{1}{3}\left(I_{\left|\right|}(t)+2I_{⊥}(t)\right)$$ (1)

and

$$r(t)=\frac{I_{\left|\right|}(t)-I_{⊥}(t)}{I_{\left|\right|}(t)+2I_{⊥}(t)},$$ (2)

with $I_{\left|\right|}(t)$ and $I_{⊥}(t)$ being the signal intensities recorded at time delay *t* for parallel and perpendicular pump/probe polarization, respectively.

For a one photon pump + one photon probe (1 + 1′) process, the anisotropy function *r*(*t*) is determined by the standard formula (3),

$$r(t)=\frac{2}{5}\left\langle \frac{3\;{\text{cos}}^{2}\left(\beta \left(t\right)\right)-1}{2}\right\rangle =\frac{2}{5}\left\langle P_{2}(\mu_{1}(0)\mu_{2}(t)\right\rangle$$ (3)

(see, e.g., the monograph by Lakowicz^46^ [Chap. 10, Eq. (10.22))]. Here, $\text{cos}(\beta (t))=\mu_{1}(0)\mu_{2}(t)$, $\mu_{1}$ and $\mu_{2}$ are the unit vectors along the TMs responsible for the transitions initiated by the pump and probe pulses, ⟨···⟩ stands for rotational averaging, and $P_{2}(x)=(3x^{2}-1)/2$ is the second order Legendre polynomial. The time dependence of $\mu_{2}(t)$ results from molecular rotation. Values for *r*(*t*) vary from initially *r*_0_ = *r*(*t = *0) = +0.4 for parallel TM orientations or *r*_0_ = −0.2 for perpendicular TM orientations to the final stationary distribution *r*(*t_dephas_*)**, where a characteristic time constant *t_dephas_* is determined by the rotational constants of the molecular system.

For a (1 + 2′) process, the anisotropy is given by a similar formula (4), which can be retrieved from Refs. 27 and 28,

$$r(t)=\frac{4}{7}\left\langle P_{2}(\mu_{1}(0)\mu_{2}(t)\right\rangle .$$ (4)

Here, we assumed that the anisotropic part of the two-photon absorption tensor is proportional to $\mu_{2}⊗\mu_{2}$, where $\mu_{2}$ is a unit vector in the molecular frame. Hence, one obtains slightly different starting values of *r*_0_ = +4/7 ≈ +0.57 and *r*_0_ = −2/7 ≈−0.29 for parallel and perpendicular TM orientations, respectively.

Single point density functional theory (DFT) and time-dependent (TD-)DFT calculations were performed at the 6–31++G(d,p) level using the ωB97XD functional^29^ within the Gaussian 09 package.^30^ Investigation of the ground (S_0_) and first excited state (S_1_) potential energy surface with respect to the dihedral angle between the xanthene and benzoic acid unit of fluorescein was performed without complete geometry optimization, as optimization of even a single excited state geometry at this level of theory is very time consuming.

## III. RESULTS AND DISCUSSION

### A. Dynamics of [FL − H]^−^

The isolated fluorescein monoanions, [FL − H]^−^ (m/z 331, most abundant isotope), were excited at their longest wavelength gas phase absorption maximum (520 nm, 0.3 *μ*J) determined in former gas phase photofragmentation studies^18,19^ and subsequently probed with high intensity pulses of longer wavelength (1200 nm, 150 *μ*J).

**FIG. 1.**
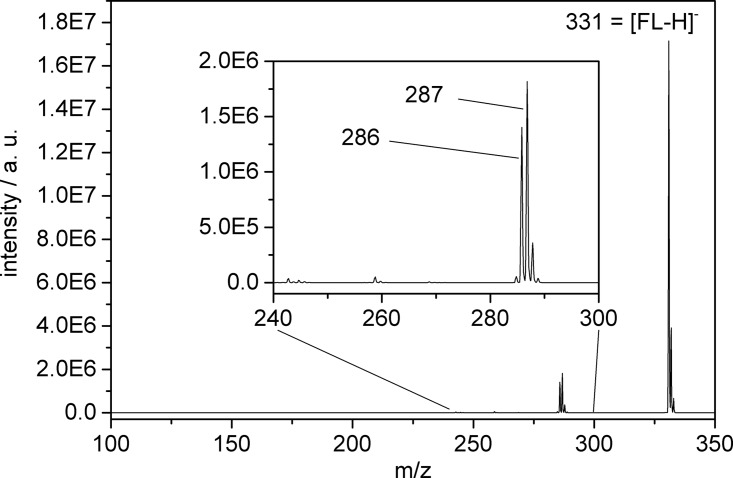
Mass spectrum depicting the formation of fragment ions after pump-probe photoexcitation of isolated [FL − H]^−^ (m/z 331, most abundant isotope).

**FIG. 2.**
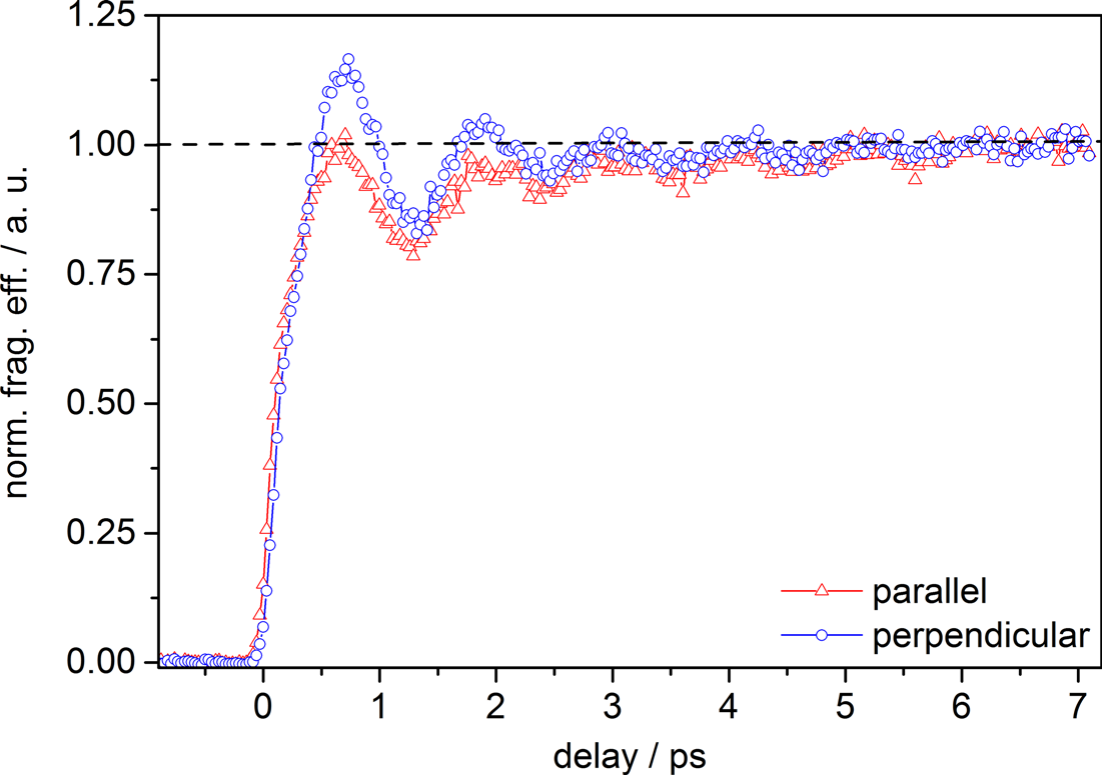
Normalized total fragmentation efficiency of [FL − H]^−^ as a function of pump-probe delay depending on the relative polarization between pump and probe pulses; λ_pump_ = 520 nm (0.3 *μ*J) and λ_probe_ = 1200 nm (150 *μ*J).

The major product fragment (for both pump-only and pump-probe photoexcitation) is located at m/z 287 or 286, corresponding to a loss of CO_2_ (- 44 Dalton) or HCO_2_ (- 45 Dalton) originating most likely from the benzoic acid moiety (Fig. 1, for a difference mass spectrum, cf. Fig. S1).^45^ As the fragmentation behavior is dominated by only one product ion, which is formed after loss of CO_2_ or HCO_2_, one can assume an efficient energy transfer from the initially excited xanthene unit to the adjacent benzoic acid moiety. Irradiation with both pump and probe laser pulses (at sub-picosecond or picosecond delay) does not lead to other additional fragmentation products but enhances the fragmentation efficiency by a factor of ca. 30 (Fig. S2)^45^ compared to fragmentation observed at negative delay (probe-pump) or by pump-only photoexcitation (Fig. 2). This signal enhancement is a prerequisite for recording tPF spectra as reported previously^23,24,31–33^ and contains information on the dynamics of the involved electronically excited states.

We performed measurements on the laser power dependence of the [FL − H]^−^ fragmentation yield for the related pump-only fragmentation and confirmed that this process is based on a multiphoton absorption (Fig. S3).^19,45^ Since the bond dissociation enthalpy for C-COOH of benzoic acid is ∼430 kJ/mol,^34^ it is conceivable that at least two photons at 520 nm (∼230 kJ/mol) are necessary for generating the observed fragment ions. However, we assume that the contribution of *multi-photon-pump + probe* photodissociation to the time-resolved signal is negligible in our experiments, as the laser intensity used (0.3 *μ*J) is very low and pump-only excitation under these conditions leads to barely noticeable fragmentation (compared to pump-probe excitation; Fig. S2).^45^ In accordance with the documented fragmentation behavior of [FL − H]^−^ in the literature,^19^ we did not observe a large contribution of a possible photodetachment loss channel. Since photodetachment leads to neutral species, their contribution to the total fragment yield is difficult to assess by ion trap methods. We note that from the difference mass spectrum (Fig. S1)^45^ one would obtain ca. 25% of the parent ion loss that cannot be accounted for by the accompanying fragment ion signal increase. This could be attributed to a photodetachment channel. However, the absolute ion signal intensities are subject to significant fluctuations of ∼10% for the parent ion and ∼15% for the main fragment ion, so that a quantitative analysis of the photodetachment yield from our data is not meaningful. Nevertheless, recently the time-resolved photodetachment of [FL − H]^−^ was studied employing 495 nm (∼242 kJ/mol) pump/400 nm (∼300 kJ/mol) probe laser pulses.^22^ The photoelectron yield under those excitation conditions was not given, but it is known (from studies on e.g., the chromophore of the green fluorescent protein)^35^ that at higher photon energies photodetachment may prevail over possible nuclear fragmentation processes. Taking these observations into account, we assume that in our case the pump (520 nm) + probe (1200 nm) signal stems from (1 + 2′) excitation, resulting in a total excitation energy of ∼430 kJ/mol, which enables fragmentation but does not lead to a large photodetachment yield.

Fig. 2 depicts the fragment ion yield as a function of time delay for parallel and perpendicular relative polarization of the pump and probe laser pulses. Signal intensities were background corrected and normalized to unity at long time delay for better comparison of the initial signal rise at time-zero and shortly afterwards. Both transients exhibit an initial rise, much slower (∼700 fs from zero to signal maximum) than the estimated system response of ∼110 fs (ccf, see Section II). However, at short time delay (0.5–4 ps), the signal recorded for perpendicular laser polarization has higher intensity than the signal recorded for parallel polarization. At longer time delay, both signals are buried in noise and virtually coincide (note: this is also true for the raw data, which are not shown and discussed here). Furthermore, both traces are sinusoidally modulated with a period of ∼1 ps. This modulation disappears within ∼6 ps. A similar dependence of a transient signal on the relative polarization orientation between pump (495 nm) and probe (400 nm) pulses, as well as a strong modulation was observed for [FL − H]^−^ in the gas-phase applying TR-PA.^22^ In TR-PA experiments, however, the total photoelectron signal intensity exhibited (1) a stronger dependence on the relative laser polarization, (2) the trace for parallel pump-probe polarization featured a higher intensity (compared to the signal at perpendicular polarization), and (3) the oscillatory component (of similar frequency) disappeared on a shorter timescale (within ∼3 ps).

**FIG. 3.**
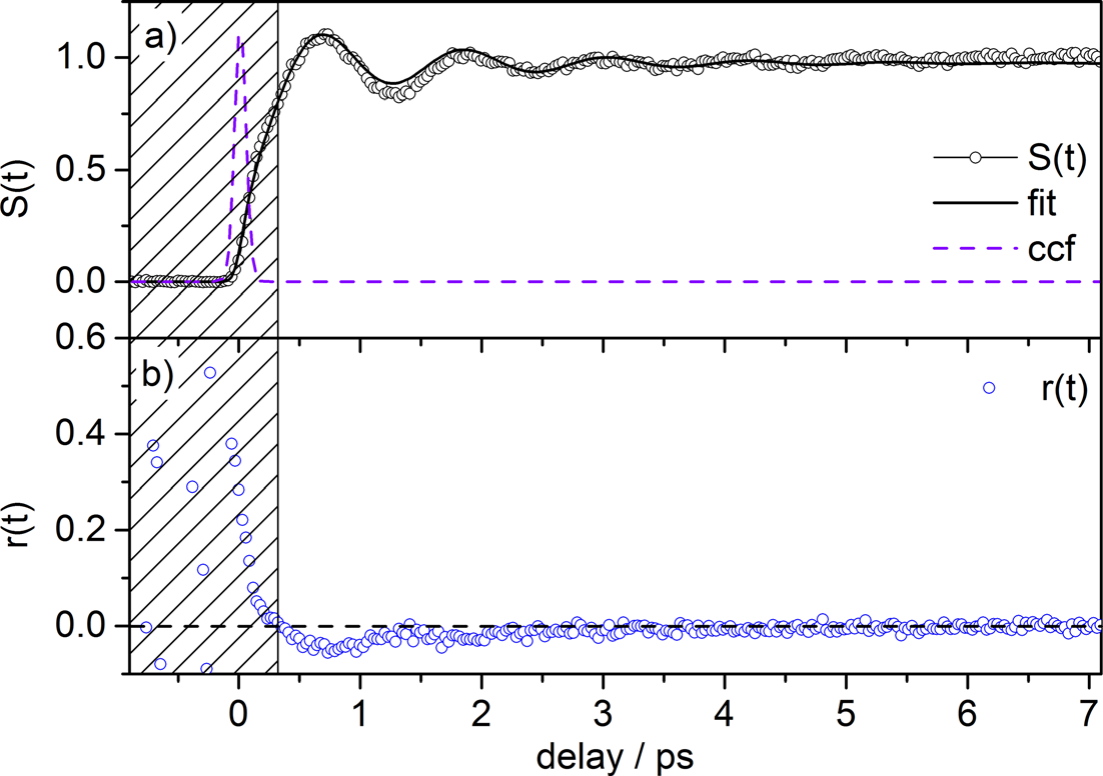
(a) Time-resolved isotropic signal function *S*(*t*) exhibiting the formation of a long lived S_1_ state superpositioned with vibrational wavepacket dynamics and (b) time-resolved anisotropy function *r*(*t*) for analysis of rotational dephasing of [FL − H]^−^; *S*(*t*) and *r*(*t*) were calculated from formulas (1) and (2), respectively.

The isotropic signal function *S*(*t*) and the anisotropy function *r*(*t*) calculated from our tPF data (Fig. 2) according to Eqs. (1) and (2) are shown in Fig. 3. Based on this decomposition analysis, as described in Section II, the trace in Fig. 3(a) depicts only the population dynamics of excited [FL − H]^−^, i.e., the preparation of a long lived (no decay was observed for a delay time of up to 800 ps) electronically excited (S_1_) state and the concurrent formation of a vibrational wavepacket, modulating the step-like transient signal. By fitting this transient to a convolution of a Gaussian with an exponentially decaying sinusoidal modulation (1+b⋅ sin(ω⋅t+ϕ)⋅ exp(−t/τ)), the oscillation period is found to be 2π/ω = T = 1.2 ps, corresponding to a vibrational frequency of 28 cm^−1^, which compares nicely to the reported frequency of 32 cm^−1^ from TR-PA experiments.^22^

The anisotropy function *r*(*t*) of a freely rotating ensemble of molecules should exhibit the dephasing of a rotational wavepacket formed upon photoselection by coherent excitation of ions (S_1_ ← S_0_) that are aligned parallel with their TMs to the pump laser polarization. Specifically, the TM for [FL − H]^−^ is oriented along the long axis of the xanthene moiety (here: the x axis, Scheme 1). The linearly polarized probe laser then samples these excited molecules by further excitation (S_n_ ← S_1_) to higher electronically excited states from which fragmentation occurs directly or after subsequent internal conversion and energy redistribution to other states. The dephasing dynamics that determines the decay of the anisotropy function *r*(*t*) from its initial value *r*_0_ to the final stationary value is determined by the temperature *T* of the ensemble (width of the rotational energy distribution or a number of states forming the rotational wavepacket) and the rotational frequencies of rotational motions of molecules perpendicular to the alignment axis, which are specified by the rotational constants or the corresponding moments of inertia. The time for reaching the minimum anisotropy value can be approximated by $t_{dephas}={\left(I_{B}/k_{B}T\right)}^{1/2}$,^36^ which yields *t_dephas_* ≈ 3 ps for fluorescein at 300 K, as recently determined in Ref. 22.

However, the experimentally determined anisotropy function for [FL − H]^−^ (Fig. 3(b)) by tPF does *not* point to a successful detection of a strong molecular alignment. First, the initial anisotropy *r*_0_ is not unambiguously obtained from our experimental data. Second, the difference in the signal for perpendicular and parallel pump-probe polarization is small. The former difficulty stems from the nearly identical and fast rise of both signals close to time zero. One should also note that the two traces for perpendicular and parallel pump-probe polarization have been measured consecutively so that we cannot exclude a slight temporal shift (∼40 fs) between the transients. This uncertainty in absolute position and thus delay time could lead to strong variations for the anisotropy function in the rising part of the signal. We therefore neglect the values for *r*(*t*) calculated in this early part of the transient (shaded area in Fig. 3(b)) and analyze only the data with *t_delay_* ≥ 300 fs. Nonetheless, a dependence on the relative laser polarization is clearly present in the data at hand, with the trace for perpendicular orientation being larger than the trace for parallel orientation (Fig. 2). Moreover, the trace recorded at magic angle relative polarization lies in its intensity between the two former scans (cf. Fig. S4).^45^ Furthermore, the difference between the two traces vanishes with increasing time delay (at ca. 4–6 ps). The function *r*(*t*) starts with small negative values (−0.05 to −0.1), which indicates perpendicularly polarized pump-probe transitions. However, theoretical (initial) values for pure transitions of that type are not matched (e.g., *r*_0_ = −0.29 for a 1 + 2′ process, see Eq. (4)). Based on the data presented, it is also difficult to estimate reliably an approximate dephasing time, as the initial anisotropy *r*_0_ could not be determined and the S/N for this data is not sufficient for a more detailed analysis. These results are different compared with the rotational dynamics for [FL − H]^−^ as obtained by TR-PA recently, which clearly point to a strong molecular alignment.^22^

### B. Dynamics of [FL + H]^+^

As our mass spectrometric setup allows for studies on both negatively and positively charged ions, we were also able to investigate and directly compare the dynamics of the related protonated fluorescein cations [FL + H]^+^ to the already discussed monoanions. This exemplifies an advantage of our ion trap gas phase approach since the separate photophysical study of [FL + H]^+^ in solution is strongly hampered by the influence of protonation equilibria.^1^

**FIG. 4.**
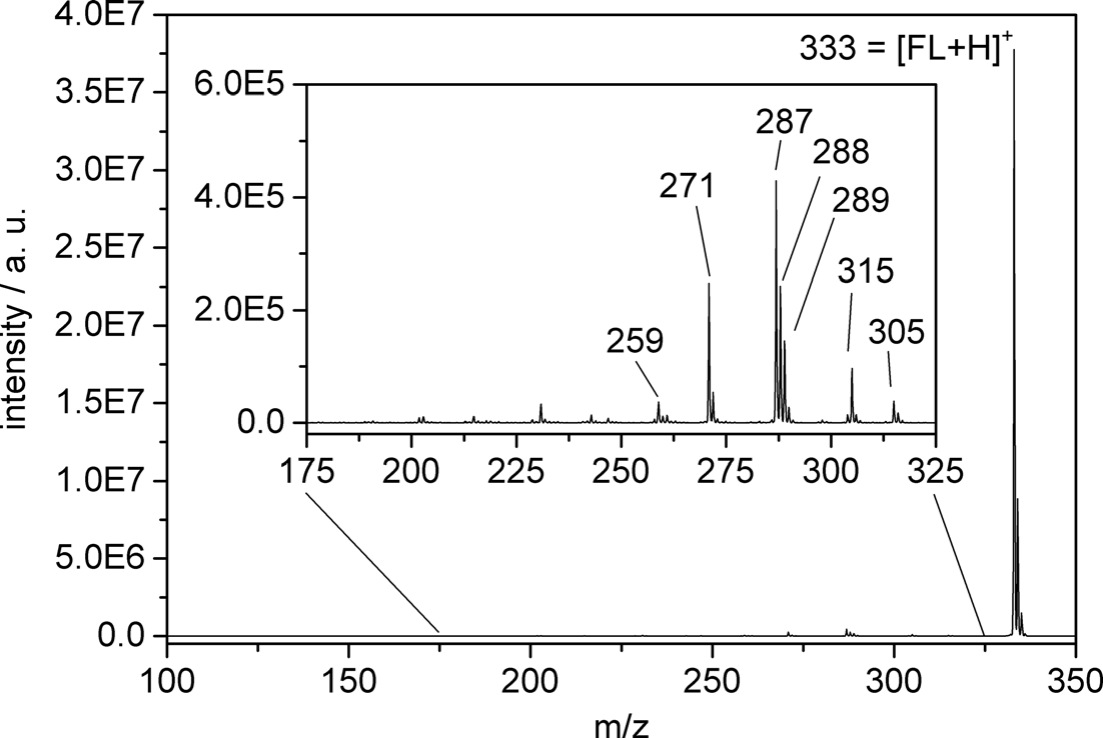
Mass spectrum depicting the formation of fragment ions after pump-probe photoexcitation of isolated [FL + H]^+^ (m/z 333, most abundant isotope).

[FL + H]^+^ ions were excited by pump laser pulses at the absorption maximum of their S_1_ ← S_0_ transition (425 nm, 0.8 *μ*J) as documented in the literature^18,19^ and subsequently probed with high intensity pulses of longer wavelength (1200 nm, 150 *μ*J). The pump-probe photofragmentation mass spectrum (Fig. 4, for a difference mass spectrum, cf. Fig. S5)^45^ of isolated [FL + H]^+^ (m/z 333, most abundant isotope) provides evidence for a more complex fragmentation behavior than observed for [FL − H]^−^. Prominent identified fragment ions are: m/z 315 (neutral loss of H_2_O); m/z 305 (neutral loss of CO); m/z 271 (neutral loss of H_2_O and HCOOH (formic acid)) apart from the main (ca. 60% of the total fragmentation signal) product ion at 287 m/z, corresponding to a loss of HCOOH, most likely from the benzoic acid moiety. This fragmentation pattern was noted before in Refs. 18 and 19 and also observed in IR multiple-photon dissociation (IRMPD) experiments.^20^ Furthermore, [FL + H]^+^ exhibits a much lower fragmentation yield than [FL − H]^−^, even when applying higher pump photon energy and intensity for photoexcitation (425 nm vs. 520 nm excitation wavelength and an intensity of 0.8 *μ*J vs. 0.3 *μ*J, respectively). Measurement of the intensity dependence of the one-color (pump-only) photofragmentation yield clearly suggests a two-photon absorption process prior to fragmentation of [FL + H]^+^ (Fig. S6).^45^ This is understandable upon consideration of the C-COOH bond dissociation enthalpy (∼430 kJ/mol for benzoic acid)^34^ vs. the pump photon energy (425 nm; ∼280 kJ/mol). Based on the much lower fragmentation yield (compared to [FL − H]^−^), we infer that this bond enthalpy is either higher for [FL + H]^+^ or energy transfer from the excited xanthene unit to the benzoic acid ring is less efficient than for [FL − H]^−^. Thus, we assume that additional dissociation channels are accessible producing a complex fragment pattern.

Interestingly, very similar fragmentation pattern was reported for both [FL + H]^+^ and [FL − H]^−^ in IRMPD experiments.^20^ This points, for both excitation schemes (nanosecond IRMPD and femtosecond UV/Vis), toward dissociation starting from highly vibrationally excited electronic ground states. However, the photonic excitation mechanisms in both cases are evidently different. Whereas the generally accepted mechanism of IRMPD is based on consecutive absorption and fast vibrational redistribution steps (usually within ps to ns),^37^ we have to assume for femtosecond two-photon UV photofragmentation a fast resonant absorption of two photons within one femtosecond laser pulse of ∼100 fs width. This is based on the assumption that collisional cooling in the ion trap takes place in between the laser pulses (millisecond time scale). Consequently, there is not enough time for energy redistribution between absorption of these photons, unless it proceeds on a sub-100 fs timescale. Subsequent internal conversion leads to highly vibrationally excited states from which eventually fragmentation takes place.

**FIG. 5.**
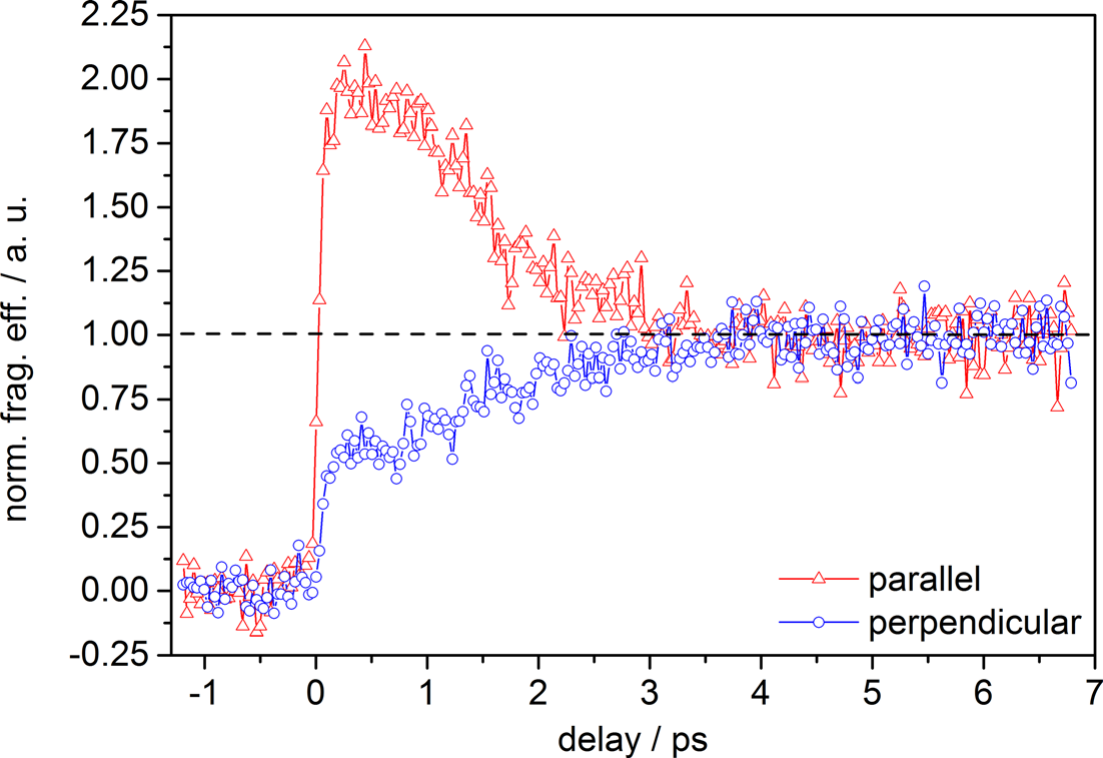
Normalized total fragmentation efficiency of [FL + H]^+^ as a function of pump-probe delay depending on the relative polarization between pump and probe pulses; λ_pump_ = 425 nm (0.8 *μ*J) and λ_probe_ = 1200 nm (150 *μ*J).

Fig. 5 depicts the total fragment ion yield of [FL + H]^+^ as a function of time delay for parallel and perpendicular pump-probe polarization. The data were background corrected and the signal was normalized to unity at long time delay, as already described for measurements on [FL − H]^−^. Comparing the results obtained for [FL − H]^−^ and [FL + H]^+^, the difference is striking. First and foremost, the transients of [FL + H]^+^ show a strong dependence on the relative laser polarization, with the transient for parallel polarized pump and probe pulses exhibiting a much stronger signal at short time delay compared to the trace for perpendicular polarization. Both polarization traces approach a constant signal value (equivalent to the magic angle signal level) after a time delay of ca. 3 ps. Secondly, the initial rise time of the transient signal for both traces is much shorter (∼250 fs) than obtained for [FL − H]^−^. Finally, no periodic modulation of the signal can be observed.

**FIG. 6.**
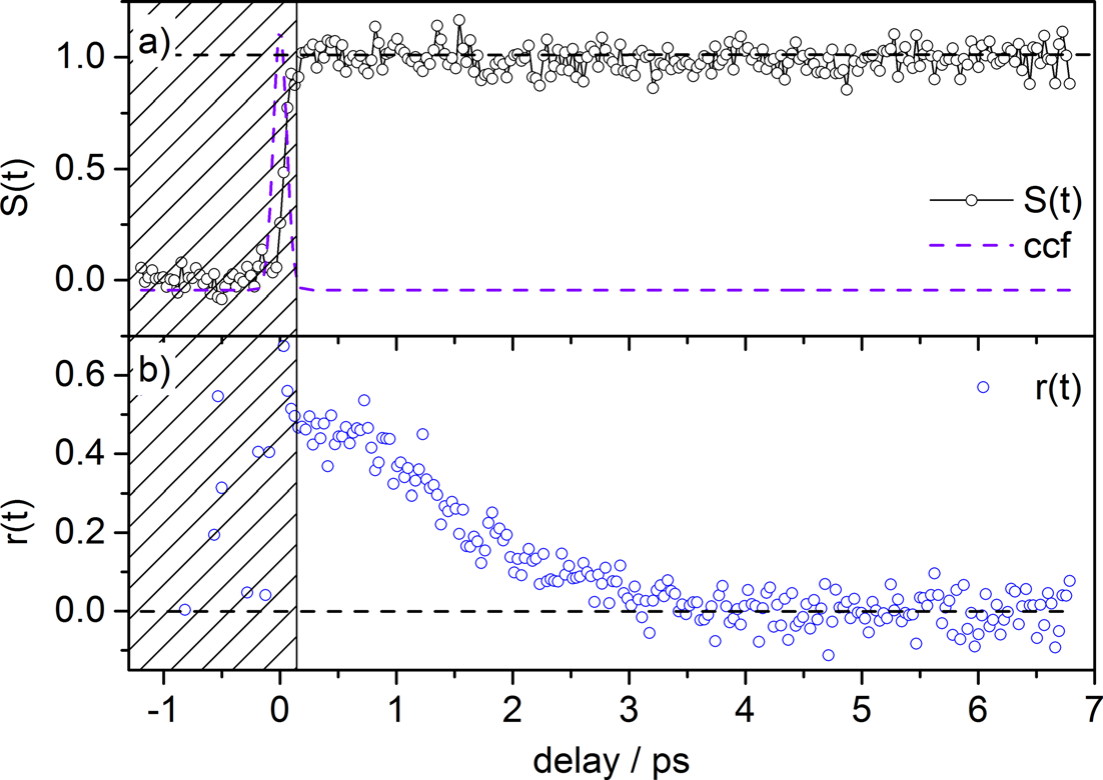
(a) Time-resolved isotropic signal function *S*(*t*) exhibiting the formation of a long lived S_1_ state and (b) time-resolved anisotropy function *r*(*t*) for the analysis of rotational dephasing of [FL + H]^+^; *S*(*t*) and *r*(*t*) were calculated from formulas (1) and (2), respectively.

For further analysis, the isotropic signal *S*(*t*) (Fig. 6(a)) and anisotropy signal *r*(*t*) (Fig. 6(b)) were calculated from the data shown in Fig. 5. By removing the contribution of rotational dephasing from the transient, it is revealed that the transient signal of [FL + H]^+^ shows no ultrafast dynamics besides rapid formation of a long lived state (similar to [FL − H]^−^, no notable decay was observed for a time delay of up to 800 ps). Furthermore, no oscillatory component and thus vibrational coherence can be discerned from the calculated isotropic signal.

From the calculated anisotropy function *r*(*t*), one can distinguish two notable features: first, the anisotropy is lost after ca. 3 ps, as the function reaches its minimum value. Second, the anisotropy has an initial value of *r*_0_ ≈ +0.5. A value for *r*_0_ exceeding +0.4 in fluorescence anisotropy investigations is usually indicative of a multiphoton excitation process, reaching a maximum of 0.57 and 0.67 for a two photon absorption and three photon absorption process, respectively (assuming a parallel orientation of the transition dipole moments for excitation and probing).^38–40^ According to Eq. (4), for example, the value of *r*_0_ ≈ +0.5 can be recovered assuming $r_{0}=(4/7)P_{2}(\mu_{1}\mu_{2})=0.5$, which yields an angle of ≈17° between $\mu_{1}$ and $\mu_{2}$. Given that the calculated rotational constants for the electronic ground states of [FL + H]^+^ and [FL − H]^−^ differ only by 1%–4% (cf. Fig. S7 and Table SI),^45^ one can estimate that the characteristic rotational decay times are very similar. Moreover, we estimate that the changes of the rotational constants by electronic excitation will be on the same order of magnitude, so that in the following we use only the ground state constants for analysis. For [FL + H]^+^, the calculated ground state rotational constants are: *A* = 0.2285 GHz, *B* = 0.2229 GHz, and *C* = 0.1384 GHz, resulting in the following moments of inertia: *I_A_* = *I_x_* = 3.673 × 10^–44 ^ kg m^2^, *I_B_* = *I_y_* = 3.765 × 10^−44 ^kg m^2^, and *I_C_* = *I_z_* = 6.064 × 10^−44 ^kg m^2^. Thus, *t_dephas_* at 300 K is estimated to ∼3 ps, as discussed for [FL − H]^−^ (see Section III A).

**FIG. 7.**
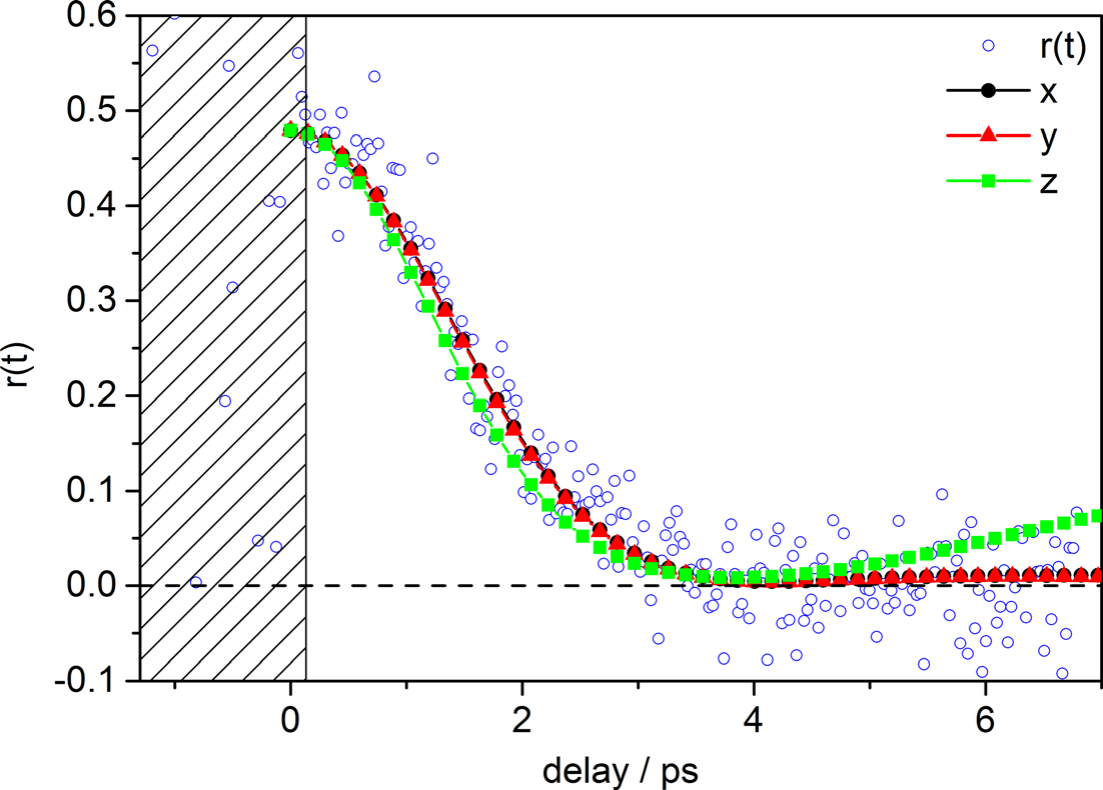
Time-resolved anisotropy *r*(*t*) exhibiting rotational dephasing of [FL + H]^+^. Black dots, red triangles, and green squares represent *r*(*t*) simulated for TMs $\mu_{1}=\mu_{2}$ directed along the axis of the smallest, intermediate and largest moment of inertia, respectively. Blue circles show experimental *r*(*t*) from Fig. 6(a).

In order to model the dynamics of the rotational anisotropy, i.e., rotational dephasing time, in more detail, we have simulated the time-resolved orientational correlation function for different TM orientations, assuming parallel polarization for both pump and probe steps ($\mu_{1}=\mu_{2}$). After scaling for the maximum value, our simulation reproduces the experimental *r*(*t*) trace nicely (Fig. 7), assuming that the TMs are parallel to the axis of the smallest moment of inertia. The theoretical basis for the simulations and their input parameters, i.e., orientation of TM, moments of inertia and temperature, are given in the Supplementary material^45^ (see also Ref. 41).

### C. Comparison and discussion

Comparing the presented experimental results for the ultrafast dynamics of the ion-trapped and isolated deprotonated fluorescein [FL − H]^−^ and protonated fluorescein [FL + H]^+^ the following points are remarkable: (1) [FL − H]^−^ exhibits upon electronic excitation strong vibrational coherences in its tPF trace whereas [FL + H]^+^ does not display this behavior, (2) [FL − H]^−^ exhibits only a weak anisotropy pointing towards perpendicularly oriented pump-probe transitions, whereas [FL + H]^+^ shows a clear and strong alignment based on parallel oriented pump-probe transitions with a characteristic rotational dephasing time. Dephasing for [FL + H]^+^ can be successfully modeled employing the ion ensemble temperature and the relevant molecular rotational constants (moments of inertia). Furthermore, (3) in comparison of our results for [FL − H]^−^ to the recently reported data by TR-PA,^22^ one can state a good agreement with respect to the observation and frequencies of the vibrational coherence (28 cm^−1^ vs. 32 cm^−1^). However, the results for the time-resolved anisotropy function are completely different. Only weak anisotropy for our employed photofragmentation scheme was found, but strong anisotropy and rotational dephasing was obtained for photoelectron detection.^22^ How can we rationalize this drastic difference in ultrafast dynamics and anisotropy of these two isoelectronic molecular systems?

**FIG. 8.**
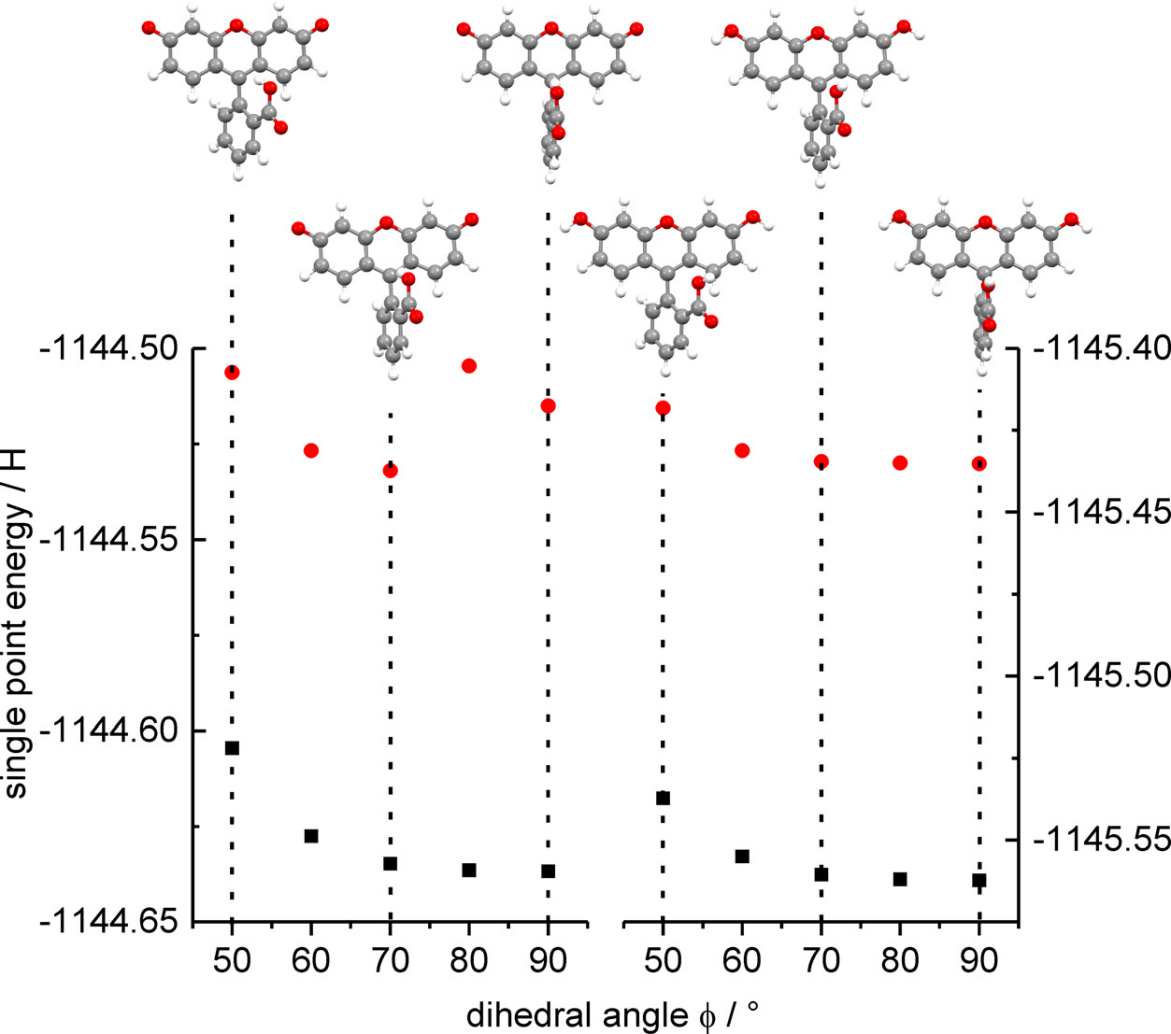
Calculated singlet point energies of the S_0_ (black squares) and S_1_ (red circles) states depending on the dihedral angle φ (cf. Scheme 1). Calculations for [FL − H]^−^ (left) suggest an excited state geometry with lower energy for φ < 90° in contrast to the energetically lowest ground state geometry (φ ≈ 90°). For [FL + H]^+^ (right), the energetically lowest S_0_ and S_1_ geometries are obtained at a dihedral angle of φ ≈ 90°.

The occurrence of vibrational coherences upon electronic excitation, i.e., preparation of vibrational wavepackets in the excited state is usually connected to an inherent vibrational excitation of the electronically excited state with respect to one or several specific modes. If the ground and excited state exhibit significantly different geometries along a vibrational coordinate, coherent excitation of several similar vibrational modes in the excited state potential energy surface is possible. A vibrational wavepacket, comprised of several superimposed vibrational states with a favorable phase relation, is then launched, which oscillates with a period close to the relevant vibrational mode and finally decays by coupling to other vibrational modes. Since the vibrational timescale is usually much shorter than the rotational one, a superposition of vibrational and rotational wavepackets may be observed, as reported, e.g., for polarized four-wave mixing spectroscopy on gaseous iodine.^42^ In the case of [FL − H]^−^, Horke *et al*. assigned a low-frequency torsional motion of the benzoic moiety vs. the xanthene unit to the experimentally observed vibration of 32 cm^−1^. The dihedral angle between both units changes from 90° in the ground state to 53° in the electronically excited state.^22^ This assignment was supported by TD-DFT calculations and geometry optimization of the first electronically excited state of [FL − H]^−^. We have observed a similar frequency (28 cm^−1^) for [FL − H]^−^ by tPF, so that its rationalization can be given on the same grounds. However, for [FL + H]^+^ this relaxation coordinate seems not to play a significant role. In order to support this hypothesis, we have performed single-point energy calculations (method see Section II) for the transition energies of distorted ground state geometries with dihedral angles between 90° and 40° (Fig. 8), which clearly show that a relaxation for [FL − H]^−^ is energetically possible whereas for [FL + H]^+^ only an increase of energy for φ < 90° was found. These theoretical results are consistent with our experimental observations.

The differences in the time-resolved anisotropy function *r*(*t*) obtained by photofragmentation and photodetachment for [FL − H]^−^ and also the differences towards the results for [FL + H]^+^ can only be qualitatively rationalized by variations in the respective higher lying electronic states. Since the excitation (pump) wavelength for [FL − H]^−^ was nearly the same in photofragmentation and photodetachment experiments, the discrepancies can only originate from different probe wavelengths (2 × 1200 nm vs. 400 nm) and detection schemes. Most crucial for interrogating molecular alignment and thus gaining information on rotational dynamics by this pump-probe method is that the probe laser absorption is connected to a transition with a well-defined orientation of the related transition dipole moment, most desirably parallel (or perpendicular) to the pump transition. Since the number and density of electronically excited states of xanthene dyes is strongly increasing in this energy region (∼4–6 eV), we assume that this condition is not generally fulfilled. However, in Ref. 22, a polarization-sensitive probe was established by detecting energy resolved electrons after time-resolved photodetachment from anions. Similar experimental schemes for the detection of rotational wavepackets have been reported before for time-resolved photoelectron imaging of neutral molecules.^43,44^ In the detection scheme that we use in our experiments, we cannot impose polarization-sensitivity due to the long timescales (*μ*s regime) involved in the generation of photofragments and their storage in the 3D ion trap. Thus, we rely on utilization of appropriate electronic transitions for the probe laser in the molecules under study. Based on these considerations, we infer that for [FL − H]^−^ the probe laser leads to excitation of several higher lying electronically excited states with mostly perpendicular orientation of TMs, whereas for the [FL + H]^+^ only one or a few states with parallel oriented TMs are singled out.

This discussion points towards weaknesses of the tPF method that one should be aware of: the lack of knowledge about the assignment of the probe laser excitation process and its (often) multiphotonic character. We assume that the cross section for absorption of the probe laser pulse is altered for different vibronic states, which is the basis for studying ultrafast electronic dynamics by this scheme. This could make the comparison of the dynamics of different species difficult since one cannot completely exclude “unfortunate” probe transitions with either very small cross sections or very similar cross sections for initial and final states of a process. This means that in the case of small cross sections the corresponding electronically excited states may be missed and for similar cross sections it may not be possible to map electronic dynamics at all. We believe that both possibilities do not apply to the investigated [FL − H]^−^ and [FL + H]^+^ species since in both cases we observed the formation of a long-lived electronically excited and detected some ultrafast dynamics on top of that. However, at the present status of our knowledge about the higher electronically excited states of the fluorescein molecular system and the tPF method, we cannot completely rule out an explanation for the non-observation of wavepacket dynamics in [FL + H]^+^, which is based on the assumption that in this case the probe laser cross section is not dependent on the torsional wave packet dynamics.

Taking into account these considerations, we can only qualitatively characterize the molecular electronic level structure of the molecular ionic species of interest. These qualitative rationalizations have to be supported in the future by accurate electronic structure and geometry calculations for the higher lying electronic states of [FL − H]^−^ and [FL + H]^+^.

Finally, we note that preliminary experiments for other fluorescein derivatives in our laboratory (e.g., 2′,7′-Dichlorofluorescein, 2′,7′-Difluorofluorescein, 5-Aminofluorescein and 5-Nitrofluorescein), gave very similar results for the deprotonated and protonated fluorescein species, respectively.

## IV. SUMMARY AND CONCLUSIONS

In summary, we have obtained by tPF the excited state vibrational and rotational dynamics of [FL − H]^−^ and [FL + H]^+^ in an ion trap at room temperature. Pronounced vibrational wavepacket motion was observed for [FL − H]^−^, which is connected to the relaxation of the dihedral angle between the benzoic acid and xanthene units in the electronically excited state, as assigned before in Ref. 22. For [FL + H]^+^ no vibrational wavepackets were observed by tPF. Since the final state of the probe transition is not known, it cannot be completely excluded that the probe laser cross section for [FL + H]^+^ might be insensitive to possible wavepacket dynamics in the primary excited state. However, TD-DFT calculations suggest for [FL + H]^+^ an excited state structure similar to the ground state with a dihedral angle of 90°, so that a wavepacket could not be launched for this torsional angle.

The polarization dependence of the tPF transients for [FL − H]^−^ was weak and could be related to a superposition of probe transitions with perpendicularly aligned TM with respect to the xanthene unit. For [FL + H]^+^ a strong polarization dependence was observed and the time-resolved anisotropy function was determined. The observed rotational dephasing was interpreted by the loss of anisotropy of an [FL + H]^+^ ion ensemble at room temperature. The dephasing of the corresponding rotational wavepacket was successfully simulated by the time-resolved orientational correlation function. From this analysis, we conclude that the probe process of [FL + H]^+^ consists of a two-photon transition with a TM aligned parallel to the xanthene unit.

In conclusion, the applied technique of femtosecond tPF in an ion trap proves to be very valuable for the analysis of ultrafast molecular vibrational and rotational dynamics and electronically excited states of ionic dye molecules under isolated conditions. It is complementary to TR-PA since it is based on the detection of fragment ions instead of the corresponding photoelectrons (for anions) and extends this methodology towards cationic systems. The drawback of the tPF method lies in the multiphotonic character of its probe process and lack of knowledge about the related absorption cross sections and the involved higher lying electronically excited states. However, the latter deficit can also be turned into a tool for gaining information on the character of these states, as demonstrated in this work.
